# Extra-Hepatic Biliary Atresia in Association with Polysplenia and Intestinal Malrotation

**Published:** 2013-10-01

**Authors:** Jaishri Ramji, Rakesh S Joshi, Mitesh Bachani, Dungarsingh Rathore

**Affiliations:** B.J.Medical College, Gujarat University, Ahmedabad, Gujarat, India

**Keywords:** Extra-hepatic biliary atresia, Polysplenia, Intestinal malrotation

## Abstract

The syndromic form of biliary atresia accounts for 10-25% and is associated with a poor prognosis due to associated anomalies. We report a case of extrahepatic biliary atresia and polysplenia syndrome with jaundice since 19th day of life and who had undergone surgical correction of malrotation in the neonatal period. Inspite of successful Kasai’s portoenterostomy at 52nd day of life, the child succumbed to post-operative sepsis.

## INTRODUCTION

Extra-hepatic Biliary atresia is a destructive inflammatory obliterative cholangiopathy of neonates and infants that affects varying lengths of the intrahepatic and extra-hepatic biliary ducts. [1] It is usually an isolated malformation, but in 10-25% cases it is associated with other anomalies. The most common association is the Polysplenia syndrome, also known as the biliary atresia splenic malformation which is reported in about 10% of cases. [1] It is a syndrome of polysplenia with extra hepatic biliary atresia and variable associations like intestinal malrotation, pre-portal duodenal vein and situs inversus. We report here a case of neonate operated for intestinal malrotation on the third day of life who then presented with cholestasis on the 19th day of life. He was then diagnosed with extra-hepatic biliary atresia and underwent Kasai procedure for the same.

## CASE REPORT

A 2-day-old first born, full-term boy, with birth weight of 3kg, born of non-consanguineous marriage with no antenatally detected anomaly presented with bilious vomiting. Maternal screens for infections were negative. His hematology and serum chemistry were within normal limits. Contrast radiogram was suggestive of malrotation (Fig. 1). Intraoperative findings confirmed malrotation and Ladd’s procedure was performed. The post-operative course was uneventful except for a prolonged period of intestinal stasis during which the nasogastric aspirate was bilious. On the eighth post-operative day, the bowel pattern became normal. When the baby was discharged on the 12th day of life, he was passing normal stools, feeding well and otherwise asymptomatic. Subsequently, on the 19th day of life, he developed jaundice and stools became acholic. On investigation, his bilirubin was 14.5mg/dl with a direct bilirubin of 5.9mg/dl suggestive of obstructive jaundice. Serum alkaline phosphatase was 243u/l and SGPT was 64u/l. Infectious and metabolic screens for evaluation of neonatal cholestasis including TORCH (toxoplasmosis, rubella, cytomegalovirus, and herpes) titers, urine culture, hepatitis B, serum alpha-1 antitrypsin and neonatal thyroid screen were negative. On ultrasonography of the abdomen, the gall bladder could not be visualized. HIDA (hepato-biliary iminodiacetic acid) scan done after priming with phenobarbitone for five days showed no clearance of tracer up to 24 hours. MRCP (magnetic resonance cholangio-pancreaticography) showed non-visualization of gall bladder and atretic extra-hepatic biliary duct with normal intrahepatic radicals on T2 weighted images. Since all these reports were suggestive of biliary atresia, we decided to perform an operative cholangiogram to confirm the diagnosis and plan definitive management.

The baby underwent exploratory laparotomy on the 52nd day of life. The gall bladder was rudimentary and fibrotic and the extra-hepatic biliary duct was obliterated (Fig. 2). The liver was firm and smooth. Polysplenia was noted with three spleens in the left hypochondrium and one splenenculus in the mesocolon (Fig. 3). After mobilizing and delivering the liver, the atretic gall bladder and portal plate were dissected. The fibrous cone at the bifurcation of the portal vein was sharply transected. A Roux-en-Y loop of proximal jejunum was brought up without going through the mesocolon to perform an end to side porto-enterostomy and intestinal continuity restored by end to side jejuno-jejunostomy 40cm beyond the transected jejunum. The inter-bowel adhesions due to the previous surgery made the dissection difficult and tedious. The initial postoperative course was stable and the baby started passing bilious stool on the fourth post-operative day, but he developed severe sepsis and succumbed on the 10th day. The liver biopsy showed cholestasis, peri-portal inflammation, bile duct proliferation and fibrosis.

**Figure:**
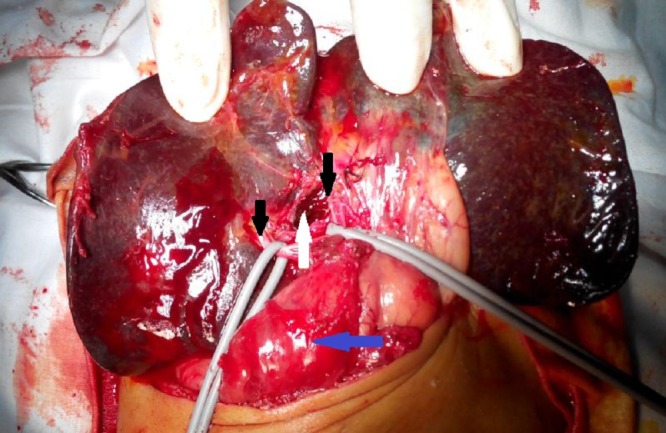
Figure 1: Intra-operative picture of portal dissection during Kasai procedure. Black arrows – right and left portal veins, White arrow – fibrous cone transected at porta, Blue arrow-Duodenum.

**Figure:**
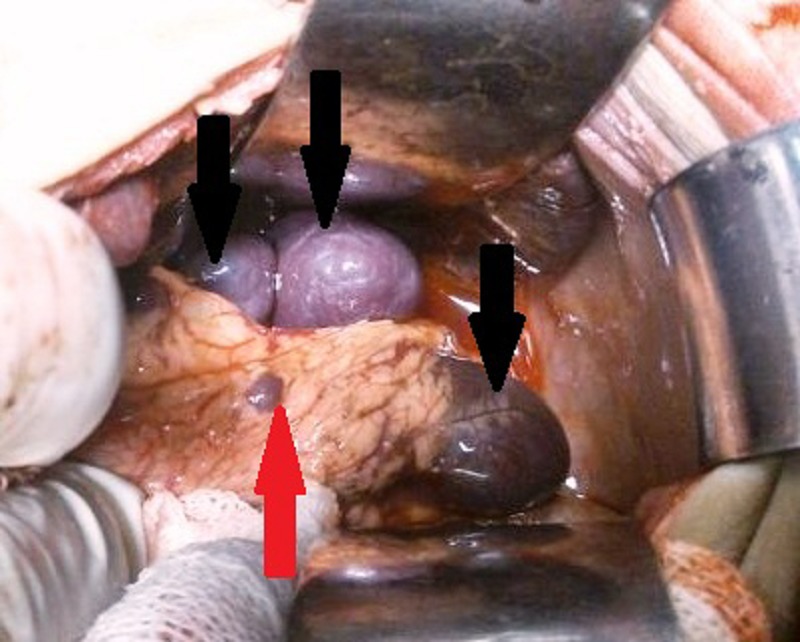
Figure 2: Intra-operative picture of polysplenia, Black arrows – multiple spleens, Red arrow – splenenculus in mesocolon.

## DISCUSSION

Biliary atresia is the end result of a destructive, idiopathic, inflammatory process that affects the intra and extra-hepatic biliary ducts leading to fibrosis and obliteration of the biliary tract and development of biliary cirrhosis. [2-3] The estimated incidence is 1 in 9000-16000 live births. [4] Two forms of the disease have been recognized; syndromic biliary atresia or the fetal or embryonal form, which is associated with other anomalies (10-35%) and non-syndromic biliary atresia or peri- or post-natal form which is an isolated anomaly (70-90%). [5]

The polysplenia syndrome or the biliary atresia splenic malformation syndrome is defined as the association of extra hepatic biliary atresia with polysplenia and a variable combination of intestinal malrotation, abdominal situs inversus, cardiac, vascular and pulmonary anomalies. Other infrequently reported associations are tracheo-esophageal fistula, renal anomalies, polydactyly, annular pancreas, choledochal cyst and intestinal atresias. [6] The consistency of the group of anatomical abnormalities forming the biliary atresia splenic malformation syndrome implies that a crucial event takes place in the genetically controlled developmental pathway at a specific stage of embryogenesis. [1] A defect in organogenesis at 40-50 days of gestation in form of an alteration in the laterality process during embryogenesis is thought to be the mechanism behind these aberrations, leading to insufficient, absent or defective development of organs on one side and excessive development on the other side. [7] 

The presence of extra hepatic anomalies and altered anatomy might be expected to adversely affect the surgical management; however studies show that in experienced centres, there is no significant difference in the surgical outcome and long term bile drainage. [7,8] The placement of Roux-en-Y loop could be challenging due to altered anatomy. In a usual case of biliary atresia, the Roux-en-Y loop is passed through a rent made in the mesentery of transverse colon; however in case of malrotation and reverse rotation of gut there could be difficulties in the orientation of the roux-en-y loop. Our patient was an unusual case where malrotation had been previously corrected before the diagnosis of extrahepatic biliary atresia. The duodenum was intraperitoneal and the roux-en y loop was directly taken to the portal plate without going through the mesocolon.

The overall prognosis of the syndromic form of biliary atresia in association with polysplenia is worse with poor bile secretion in comparison to the non-syndromic variety. One of the factors for this may be the imprecise identification of porta hepatis during surgery. [9] The biliary atresia splenic malformation syndrome has been shown to be one of the prognostic factors for an overall poor outcome in patients of biliary atresia. It is unclear whether the reason for this is the presence of other associated anomalies which are themselves detrimental. [10-13]. Moreover, in these patients, increasing age has a significant detrimental effect on the outcome. [13]

EHBA is a rare disease with complete obstruction of bile flow that develops as a result of sclerosing fibro-obliteration of the extrahepatic bile duct. Syndromic type of the disease is very rare and although the surgical outcome is similar to the non-syndromic variety, the overall prognosis is worse due to other associated anomalies. 

## Footnotes

**Source of Support:** Nil

**Conflict of Interest:** None

